# Solitary fibrous tumor resembling pulmonary fractionation disease: A case report

**DOI:** 10.1097/MD.0000000000034290

**Published:** 2023-07-07

**Authors:** Ryusei Yoshino, Nana Yoshida, Akane Ito, Masaki Nakatsubo, Sayaka Yuzawa, Masahiro Kitada

**Affiliations:** a Department of Thoracic Surgery and Breast Surgery, Asahikawa Medical University Hospital, Asahikawa-shi, Hokkaido, Japan; b Department of Diagnostic Pathology, Asahikawa Medical University Hospital, Asahikawa-shi, Hokkaido, Japan.

**Keywords:** clinical utility, clinimetric properties, paresis, physical assessment, strength testing

## Abstract

Preoperative differentiation between pulmonary fractionation and solitary fibrous tumors (SFTs) is challenging. Diaphragmatic primary tumors are relatively rare among SFTs, with limited reports of abnormal vascularity.

**Patient concerns:**

A 28-year-old male patient was referred to our department for surgical resection of a tumor near the right diaphragm, Thoracoabdominal contrast-enhanced computed tomography (CT) scan revealed a 10 × 8 cm mass lesion at the base of the right lung. The inflow artery to the mass was an anomalous vessel in which the left gastric artery bifurcated from the abdominal aorta, and its origin was the common trunk and right inferior transverse artery.

**Diagnosis:**

The tumor was diagnosed as right pulmonary fractionation disease based on the clinical findings. The postoperative pathological examination determined a diagnosis of SFT.

**Interventions:**

The pulmonary vein was used to irrigate the mass. The patient was diagnosed with pulmonary fractionation and underwent surgical resection. Intraoperative findings revealed a stalked, web-like venous hyperplasia anterior to the diaphragm, contiguous with the lesion. An inflow artery was found at the same site. The patient was subsequently treated using a double ligation technique. The mass was partially contiguous with S10 in the right lower lung and stalked. An outflow vein was identified at the same site, and the mass was removed using an automatic suture machine.

**Outcomes:**

The patient received follow-up examinations that involved a chest CT scan every 6 months, and no tumor recurrence was reported during 1 year of postoperative follow-up.

**Lessons:**

Differentiating between SFT and pulmonary fractionation disease may be challenging during preoperative diagnosis; therefore, aggressive surgical resection should be considered as SFTs may be malignant. Identification of abnormal vessels using contrast-enhanced CT scans may be effective in reducing surgical time and improving the safety of the surgical procedure.

## 1. Introduction

Pulmonary fractionation disease is an abnormal lung tissue that is vascularized from abnormal vessels of the major circulatory system and has no traffic with normal bronchi.^[1]^ Conversely, solitary fibrous tumors (SFTs), particularly those of diaphragmatic origin, are relatively rare mesenchymal tumors with a predilection for the pleura.^[2]^ It is reportedly difficult to differentiate between the 2 conditions,^[3]^ and the identification of abnormal blood vessels is often difficult. In the present case, abnormal blood vessels were identified preoperatively using contrast-enhanced computed tomography (CT) scans, and the patient was diagnosed with pulmonary fractionation disease. However, histopathological examination following surgical resection revealed a SFT. We report a case in which preoperative identification of abnormal blood vessels in the lung tissue allowed us to perform a safe and rapid surgical tumor resection.

## 2. Case presentation

The patient was a 28-year-old male diagnosed with an abnormal chest shadow during a medical checkup in 2022. No abnormalities were noted in past physical examinations. A chest CT scan revealed a tumor near the right diaphragm, which was diagnosed as right lung fractionation disease, and the patient was referred to our department for surgical resection of the tumor.

The patient had no significant family history of disease, including no family history or diagnosis of cardiac disease. The patient had a history of allergic rhinitis but reported no previous history of smoking or alcohol consumption. The patient characteristics at admission were as follows: The patient was 176 cm tall, weighed 58 kg, and had a body mass index of 18.7. No abnormal respiratory sounds were noted in the chest. No cervical lymph node enlargement was observed. Laboratory findings at admission reported no abnormal findings in blood count, biochemistry, or coagulation. The tumor markers, CEA (1.3 mg/mL) and CA19-9 (8.5 U/mL) were not elevated. There were no abnormal findings on respiratory function or electrocardiography.

**Figure 1.**
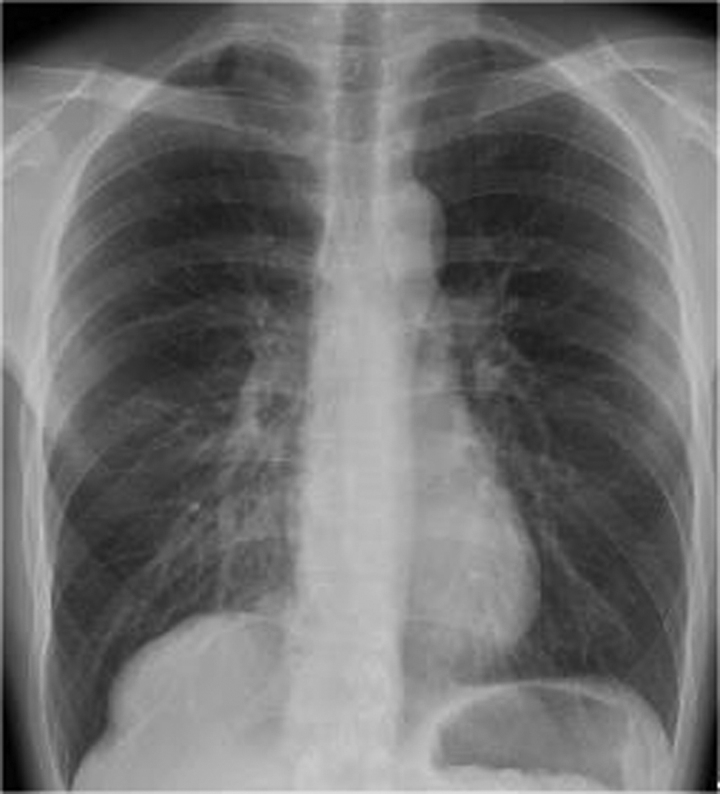
Chest radiograph (frontal view). The right diaphragmatic region shows a tumorous lesion.

**Figure 2.**
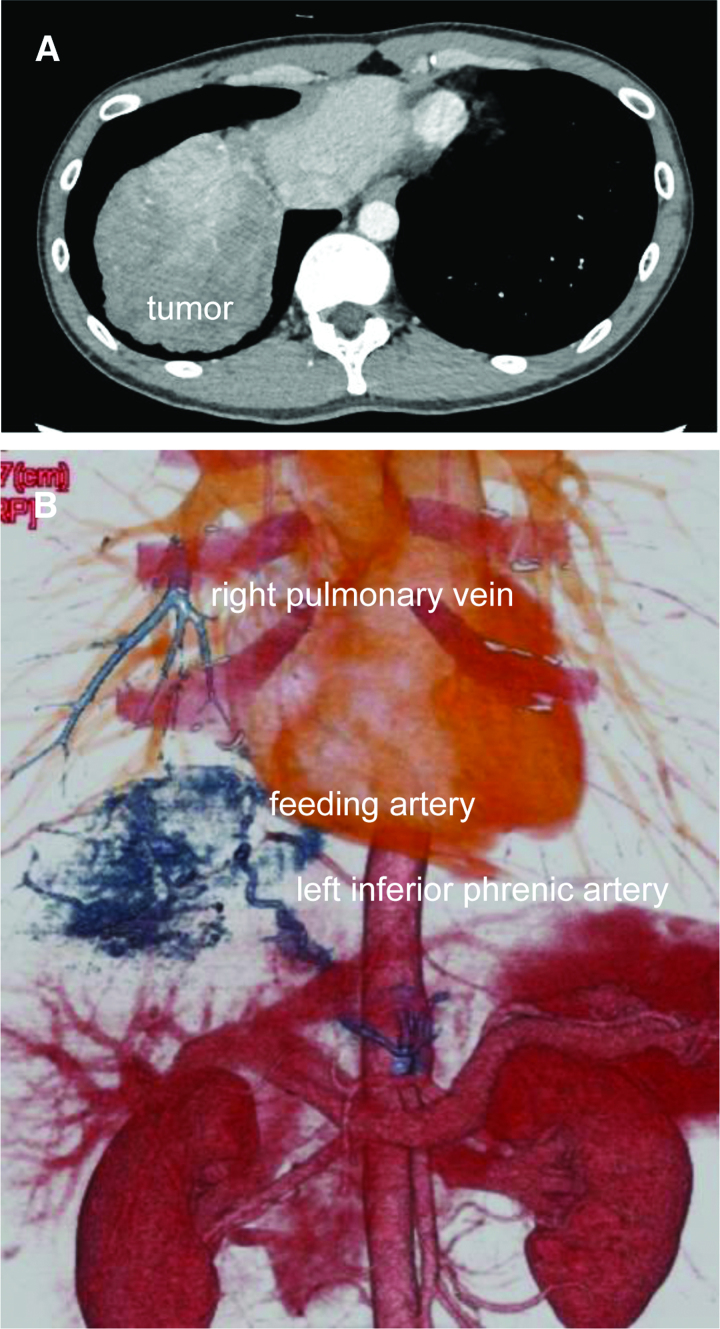
Chest computed tomography (CT) findings. (A) A tumorous lesion measuring 10 × 8 mm was identified in the right lung base. (B) The feeding artery to the tumor was found to branch off from the abdominal aorta. An anomalous vessel was observed branching from the abdominal aorta and sharing a common trunk with the left gastric artery, which in turn branched off from the anomalous vessel. The left inferior phrenic artery was found to branch off from the left gastric artery. The venous drainage from the tumor was flowing into the right pulmonary vein.

Chest radiography images revealed a mass on the right side of the diaphragm (Fig. 1). A contrast-enhanced CT scan of the thorax and abdomen revealed a 10 × 8 cm mass lesion at the base of the right lung (Fig. 2A). The artery inflow to the mass was a branch of the abdominal aorta. The left gastric artery was an anomalous vessel branching from the abdominal aorta and was the common trunk of the inflow artery. The left inferior transverse artery diverged from the left gastric artery (Fig. 2B). Three-dimensional (3D) reconstruction showed normal bronchial bifurcation. A bronchoscopy was not performed. The patient was finally diagnosed with pulmonary fractionation disease. The patient was asymptomatic; however, at the patient’s request, surgical resection was performed at an early stage.

**Figure 3.**
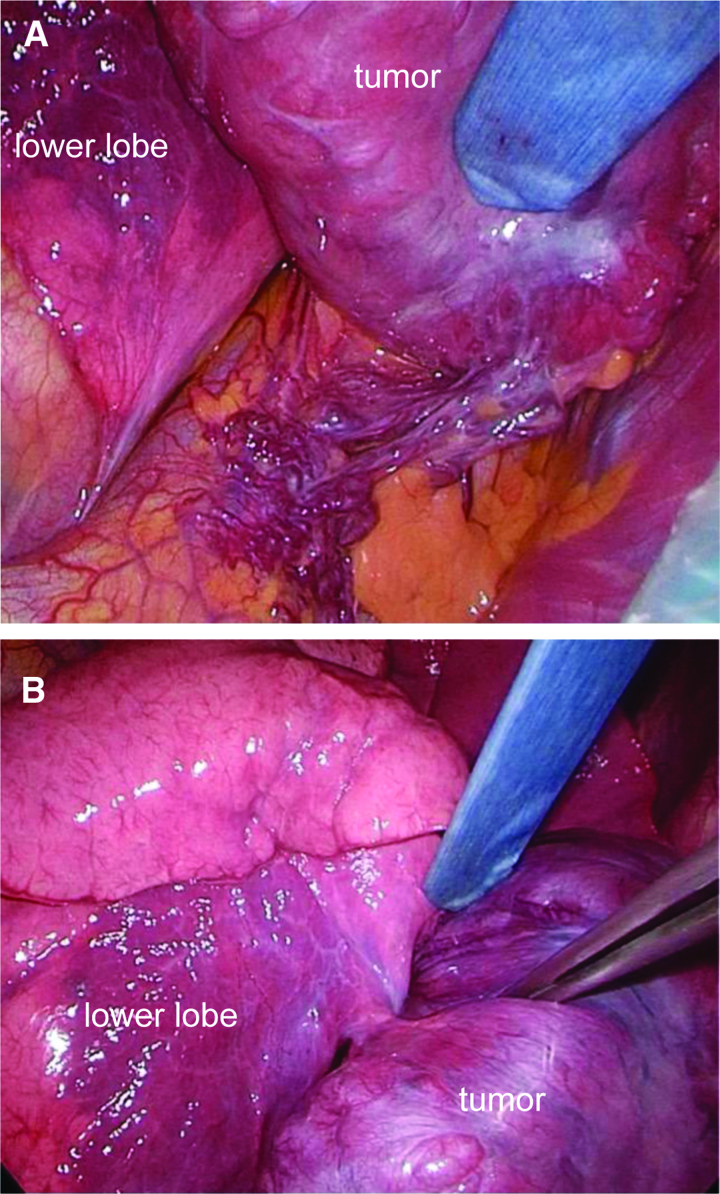
Intraoperative findings. (A) Web-like venous malformation was identified in the anterior part of the diaphragm, which was continuous with the lesion and pedunculated. An inflow artery was judged to be present in the same area. (B) The tumor was partially continuous with the right lower lung S10 and was pedunculated. A venous outflow was judged to be present in the same area.

The intraoperative findings were recorded for the surgical resection procedure. A small thoracoscopically assisted right fifth intercostal chest incision was made. Intrathoracic examination revealed no obvious adhesions to the diaphragm. Web-like venous hyperplasia was observed anterior to the diaphragm, which was contiguous with the lesion and stem-like (Fig. 3A). The mass was partially treated using a double ligation technique. Further, the mass was partially contiguous with S10 in the right lower lung and stalked (Fig. 3B). An outflow vein was present in the same area, and the mass was removed using an automatic suture machine. The thoracic cavity was cleaned, and a leak test was performed. The mean operative time was 43 minutes, and the bleeding volume was 10 mL.

**Figure 4.**
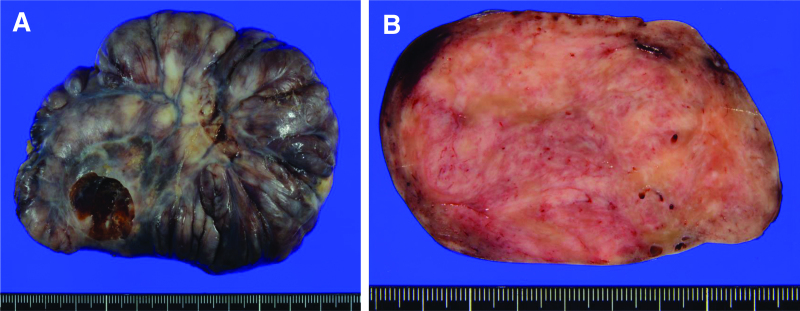
Findings of the resected specimen. (A) The specimen was 11 × 9 × 5 cm in size, and apart from the vascular dissection at the lung base, it was entirely covered by the pleura. (B) The cut surface of the tumor was tan, multinodular, and firm.

**Figure 5.**
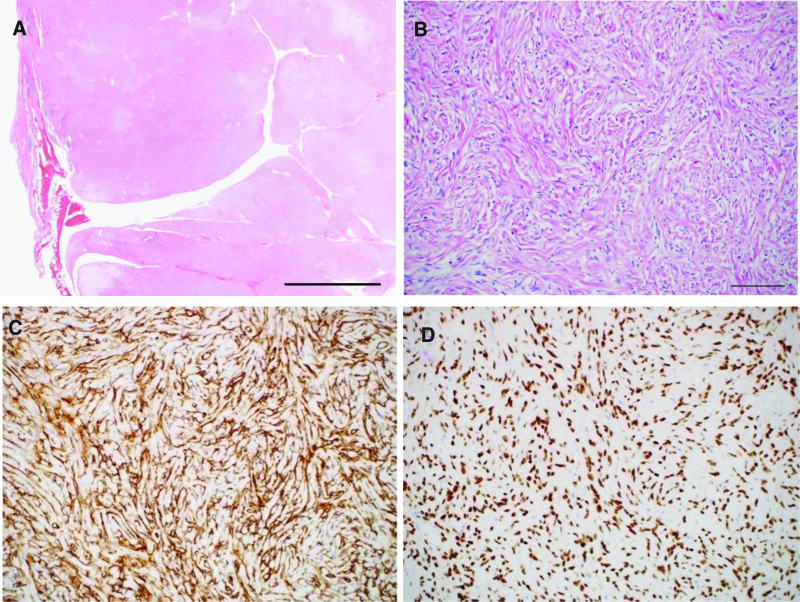
Histopathological findings. (A) The tumor was well-demarcated and showed solid growth or a leaf-like pattern. (B) The tumor showed a patternless or storiform growth of spindle cells with collagenous stroma. No mitotic figures or necrosis was observed (hematoxylin-eosin [HE] staining, Scale bar: (A) 5 mm, (B) 100 μm). (C) The tumor cells were positive for CD34. (D) The tumor cells were immunoreactive for STAT6. STAT6 = signal transducer and activator of transcription-6.

The resected specimen was 11 × 9 × 5 cm in size, and the pleura covered the entire specimen except for the vasectomy at the base of the lung (Fig. 4A). The cut surface was tan, multinodular, and firm (Fig. 4B). Histopathologically, the tumor was well-demarcated and composed of uniform spindle cells in a patternless or a storiform pattern with collagenous stroma (Fig. 5A and B). Mitotic activity, pleomorphism, and necrosis were absent. At the periphery, the tumor infiltrated into the lung parenchyma and showed a leaf-like growth pattern reminiscent of a phyllodes tumor with entrapment of respiratory epithelium (Fig. 5B). Immunohistochemistry revealed that the spindle-shaped cells were positive for CD34 (Fig. 5C) and signal transducer and activator of transcription-6 (STAT6) (Fig. 5D). Other proteins, such as alpha-smooth muscle actin, desmin, and S100 proteins, were not expressed, and the MIB-1 index was approximately 3%. Based on these findings, SFT was diagnosed, and the patient was classified as low-risk according to the WHO 5th Risk Classification. No evident venous or lymphatic invasion and draining veins were observed, and resection margins were negative. Postoperative adjuvant therapy was not administered. The patient received follow-up examinations which involved a chest CT scan every 6 months, and no tumor recurrence was reported during 1 year of postoperative follow-up.

## 3. Discussion

Pulmonary fractionation disease is defined as an abnormal lung tissue that exists in isolation from the normal lung and is vascularized by an abnormal blood supply from the general circulation system.^[4]^ Pulmonary fractionation disease can be categorized as intralobular lung fractionation syndrome, in which the fractionated lung is covered by a common pleura with the normal lung, and extrapulmonary lung fractionation syndrome, in which the fractionated lung is not covered by a common pleura. The diagnosis of pulmonary fractionation requires the identification of abnormal arteries,^[5]^ which is often difficult owing to their small diameters. Moreover, it is difficult to distinguish pulmonary fractionation from mediastinal tumors or SFTs.^[3]^ Previous reports have indicated that a preoperative diagnosis of pulmonary fractionation disease favors a more conservative treatment approach due to the risk of damaging abnormal blood vessels during surgical intervention.^[6]^ Nevertheless, surgical resection is frequently performed to differentiate SFT from other diseases, as described earlier.^[7]^

An SFT is a rare tumor of mesenchymal origin which typically originates within the thoracic cavity, predominantly from the pleura; however, other extrathoracic origin sites have also been identified, including the head and neck, abdomen, pelvis, extremities, meninges, and scrotum.^[8]^ SFTs with hypoglycemia have also been reported.^[9,10]^ Histopathologically, SFTs comprise spindle-shaped cells with fibroblast-like round or oval nuclei that grow in an unstructured and disorganized manner.^[11,12]^ Immunohistological features include strong expression of CD34, CD99, and BCL-2 in > 90% of cases.^[13,14]^ The genetic hallmark of SFT is the *NAB2:: STAT6* gene fusion and immunostaining with anti-STAT6 antibodies have been reported to be useful for the diagnosis of SFT.^[14]^ STAT6 immunostaining is the most sensitive and specific marker currently available in several studies. England et al^[15]^ defined the criteria for a malignant SFT as mitotic figures of 4/10 HPF or more; increased cell density; nuclear hyperplasia; or the presence of hemorrhage or necrosis. Although several studies have been conducted using other criteria, most reports to date have relied on this definition.

In the present case, the inflow artery was identified as the left diaphragmatic artery and the perfusion vein was identified as the pulmonary vein by 3D reconstruction using a preoperative contrast-enhanced CT scan. An angiography is performed in cases where it is difficult to identify the abnormal blood vessels but is not always conducted due to the cost of medical care and the widespread use of contrast-enhanced CT scans.^[16]^ In the present case, the left gastric artery branched directly from the abdominal aorta, and the right diaphragmatic artery branched from its common trunk and was further identified as the inflow artery. The perfusion vein was identified as the pulmonary vein. Therefore, abnormal blood vessels were determined using contrast-enhanced CT imaging preoperatively, and a final diagnosis of pulmonary fractionation disease was determined. Although the patient had no subjective symptoms, surgical resection was performed upon request. The inflow artery was noted as a web-like vascular-transected area at the base of the lung; however, the outflow vein could not be clearly identified.

Intraoperative vascular treatment was straightforward as the preoperative contrast-enhanced CT images revealed abnormal vasculature. The inflow and right diaphragmatic arteries were dissected using a double ligation technique. The pulmonary vein, thought to be the perfusion vein, was partially severed as a single lump with the lung using an automatic suture machine. This case was diagnosed postoperatively as an SFT on histopathological examination. We believe that this case of SFT with such abnormal vascular motion is very rare in the literature and is therefore a valuable case study for both researchers and clinical professionals. However, it has already been suggested that postoperative histopathological examination of SFTs may show abnormal vascular motion.^[17]^ Therefore, SFTs should be considered in the preoperative evaluation of tumors with abnormal vascularity.

In this case, pulmonary fractionation was diagnosed preoperatively, and surgical resection was performed. As mentioned previously, pulmonary fractionation can be difficult to distinguish from other diseases, including SFTs. In this case, the SFT was diagnosed as benign; however, a malignant SFT should have been considered. A biopsy may be beneficial for a definitive preoperative diagnosis. However, in the case of malignant SFTs, needle tract seeding has been previously reported and is known to cause tumor recurrence in the puncture route.^[18]^ Therefore, the indication for biopsy should be carefully considered. Therefore, aggressive surgical resection should be prioritized in all cases.

## 4. Conclusion

We report a relatively rare case, wherein anomalous vessels were identified preoperatively, and a diagnosis of pulmonary fractionation was determined. However, histopathological examination revealed an SFT. This case showed abnormal vascular distribution in the right diaphragmatic artery and pulmonary vein as a perfusion vein. Preoperative contrast-enhanced CT imaging enabled us to identify the vascular architecture and perform surgical resection of the tumor safely and promptly. Pulmonary fractionation disease is often difficult to diagnose preoperatively, and surgery is a useful alternative for obtaining a definitive diagnosis.

## Author contributions

**Conceptualization:** Ryusei Yoshino.

**Supervision:** Ryusei Yoshino, Nana Yoshida, Akane Ito, Masaki Nakatsubo, Sayaka Yuzawa, Masahiro Kitada.

**Validation:** Ryusei Yoshino, Nana Yoshida, Akane Ito, Masaki Nakatsubo, Sayaka Yuzawa, Masahiro Kitada.

**Visualization:** Ryusei Yoshino, Nana Yoshida, Akane Ito, Masaki Nakatsubo, Sayaka Yuzawa, Masahiro Kitada.

**Writing – original draft:** Ryusei Yoshino.

**Writing – review & editing:** Ryusei Yoshino, Sayaka Yuzawa, Masahiro Kitada.

## References

[1] Chakraborty RK Modi P Sharma S. Pulmonary sequestration. In: StatPearls. Treasure Island (FL): StatPearls Publishing 2022 July 25. 2023. 30335347

[2] Ge W Yu DC Jiang CP. Giant solitary fibrous tumor of the diaphragm: a case report and review of literature. Int J Clin Exp Pathol. 2014;7:9044–9. 25674285 PMC4314007

[3] Kaul P Kay S Gaines P. Giant pleural fibroma with an abdominal vascular supply mimicking a pulmonary sequestration. Ann Thorac Surg. 2003;76:935–7. 12963238 10.1016/s0003-4975(03)00553-8

[4] Pryce DM. Lower accessory pulmonary artery with intralobar sequestration of lung; a report of seven cases. J Pathol Bacteriol. 1946;58:457–67. 20283082

[5] Savic B Birtel FJ Tholen W. Lung sequestration: report of seven cases and review of 540 published cases. Thorax. 1979;34:96–101. 442005 10.1136/thx.34.1.96 PMC471015

[6] Robson VK Shieh HF Wilson JM. Non-operative management of extralobar pulmonary sequestration: a safe alternative to resection? Pediatr Surg Int. 2020;36:325–31. 31707604 10.1007/s00383-019-04590-2

[7] Liu C Pu Q Ma L. Video-assisted thoracic surgery for pulmonary sequestration compared with posterolateral thoracotomy. J Thorac Cardiovasc Surg. 2013;146:557–61. 23773788 10.1016/j.jtcvs.2013.04.027

[8] Nomura T Satoh R Kashima K. A case of large solitary fibrous tumor in the retroperitoneum. Clin Med Case Rep. 2009;2:21–5. 24179368 10.4137/ccrep.s2356 PMC3785379

[9] Kitada M Yasuda S Takahashi N. Non-islet cell tumor hypoglycemia caused by intrathoracic solitary fibrous tumor: a case report. J Cardiothorac Surg. 2016;11:1–4. 27061182 10.1186/s13019-016-0463-6 PMC4826510

[10] Yanagiya M Matsumoto J Miura T. Extended thoracotomy with subcostal incision for giant solitary fibrous tumor of the diaphragm. AME Case Rep. 2017;1:8. 30263995 10.21037/acr.2017.11.02 PMC6155638

[11] Hunt I Ewanowich C Reid A. Managing a solitary fibrous tumour of the diaphragm from above and below. ANZ J Surg. 2010;80:370–1. 20557515 10.1111/j.1445-2197.2010.05282.x

[12] Gold JS Antonescu CR Hajdu C. Clinicopathologic correlates of solitary fibrous tumors. Cancer. 2002;94:1057–68. 11920476

[13] Yamada Y Kohashi K Kinoshita I. Histological background of dedifferentiated solitary fibrous tumour. J Clin Pathol. 2022;75:397–403. 33975913 10.1136/jclinpath-2020-207311

[14] Yuzawa S Nishihara H Wang L. Analysis of NAB2-STAT6 gene fusion in 17 cases of meningeal solitary fibrous tumor/hemangiopericytoma. Am J Surg Pathol. 2016;40:1031–40. 26927892 10.1097/PAS.0000000000000625

[15] England DM Hochholzer L McCarthy MJ. Localized benign and malignant fibrous tumors of the pleura. A clinicopathologic review of 223 cases. Am J Surg Pathol. 1989;13:640–58. Erratum in: Am J Surg Pathol 1991;15(8):818. 2665534 10.1097/00000478-198908000-00003

[16] Xuan HY Shi KH Gong WH. Extralobar pulmonary sequestration in a 55-year-old man. Ann Thorac Cardiovasc Surg. 2014;20:564–6. 23803531 10.5761/atcs.cr.12.01997

[17] Regezi JA. Odontogenic cysts, odontogenic tumors, fibroosseous, and giant cell lesions of the jaws. Mod Pathol. 2002;15:331–41. 11904346 10.1038/modpathol.3880527

[18] Kattach H Hasan S Clelland C. Seeding of stage I thymoma into the chest wall 12 years after needle biopsy. Ann Thorac Surg. 2005;79:323–4. 15620969 10.1016/j.athoracsur.2003.08.004

